# An L213A variant of β-glycosidase from *Sulfolobus solfataricus* with increased α-L-arabinofuranosidase activity converts ginsenoside Rc to compound K

**DOI:** 10.1371/journal.pone.0191018

**Published:** 2018-01-11

**Authors:** Ji-Hyeon Choi, Kyung-Chul Shin, Deok-Kun Oh

**Affiliations:** Department of Bioscience and Biotechnology, Konkuk University, Seoul, Republic of Korea; Inha University, REPUBLIC OF KOREA

## Abstract

Compound K (C-K) is a crucial pharmaceutical and cosmetic component because of disease prevention and skin anti-aging effects. For industrial application of this active compound, the protopanaxadiol (PPD)-type ginsenosides should be transformed to C-K. β-Glycosidase from *Sulfolobus solfataricus* has been reported as an efficient C-K-producing enzyme, using glycosylated PPD-type ginsenosides as substrates. β-Glycosidase from *S*. *solfataricus* can hydrolyze β-d-glucopyranoside in ginsenosides Rc, C-Mc_1_, and C-Mc, but not α-l-arabinofuranoside in these ginsenosides. To determine candidate residues involved in α-l-arabinofuranosidase activity, compound Mc (C-Mc) was docking to β-glycosidase from *S*. *solfataricus* in homology model and sequence was aligned with β-glycosidase from *Pyrococcus furiosus* that has α-l-arabinofuranosidase activity. A L213A variant β-glycosidase with increased α-l-arabinofuranosidase activity was selected by substitution of other amino acids for candidate residues. The increased α-l-arabinofuranosidase activity of the L213A variant was confirmed through the determination of substrate specificity, change in binding energy, transformation pathway, and C-K production from ginsenosides Rc and C-Mc. The L213A variant β-glycosidase catalyzed the conversion of Rc to Rd by hydrolyzing α-l-arabinofuranoside linked to Rc, whereas the wild-type β-glycosidase did not. The variant enzyme converted ginsenosides Rc and C-Mc into C-K with molar conversions of 97%, which were 1.5- and 2-fold higher, respectively, than those of the wild-type enzyme. Therefore, protein engineering is a useful tool for enhancing the hydrolytic activity on specific glycoside linked to ginsenosides.

## Introduction

Ginseng (*Panax ginseng* C. A. Meyer) belongs to the *Panax* genus of the *Araliaceae* family and has been used as a traditional medicine in Korea, China, and Japan for thousands of years [1]. Ginsenosides are the main active constituent found in ginseng and possess anti-cancer, anti-fatigue, anti-inflammatory, anti-oxidative, anti-viral, morphine-dependence attenuating, neuroprotective, and vasorelaxative properties [2–7]. Ginsenosides are divided into the protopanaxadiol (PPD) and protopanaxatriol (PPT) types, comprising of sugars linked to the dammarane (tetracyclic triterpene) skeleton. PPD-type ginsenosides contain no sugar, one, or two β-d-glucopyranoses linked to C-3; and no sugar, inner β-d-glucopyranose, or outer α-l-arabinopyranose, α-l-arabinofuranose, or β-d-xylopyranose linked to C-20 [8]. PPT-type ginsenosides contain no sugar, inner β-d-glucopyranose, or outer β-d-glucopyranose, β-d-xylopyranose, or β-d-rhamnopyranose linked to C-6; and no sugar or β-d-glucopyranose linked to C-20. Ginsenosides (Rb_1_, Rb_2_, Rc, Rd, Rg_1_, and Re) in wild ginseng was glycosylated forms contains more than 80%, however, deglycosylated forms in ginseng are present at low concentrations or do not exist. Deglycosylated forms shows greater biological and pharmaceutical activities than glycosylated forms owing to the higher bioavailability and better absorption in the gastrointestinal tract [9].

Compound K (C-K, 20-*O*-β-d-glucopyranosyl-20(*S*)-protopanaxadiol) is one molecule of β-d-glucopyranose linked to C-20 in the dammarane skeleton and can be produced from the hydrolysis of glycosylated PPD-type ginsenosides such as Rb_1_, Rb_2_, and Rc. C-K has demonstrated beneficial pharmaceutical properties such as anti-allergic, anti-arthritic, anti-carcinogenic, anti-diabetic and anti-inflammatory activities [6, 10–16]. C-K has also exhibited beneficial cosmetic properties such as the alleviation of skin wrinkles and xerosis [17]; and the prevention of UV-induced skin photo-aging and burn-wound healing [18–20]. Therefore, C-K has been used as an essential ingredient in cosmetics and traditional medicine. Diverse methods, such as heating, acid hydrolysis, alkali treatment, and microbial and enzymatic transformation, for obtaining C-K have been carried out due to the absence of C-K in ginseng [21]. Chemical production methods such as acid and alkali treatments induce environmental pollution, while heating method result in low selectivity and yield. In contrast, enzymatic or microbial transformation of C-K from glycosylated PPD-type ginsenosides in ginseng produces no environmental pollution with highly selective hydrolysis.

β-Glycosidase from *Sulfolobus solfataricus* has been an efficient C-K-producing enzyme using glycosylated PPD-type ginsenosides as substrates because of its broad hydrolysis activity, including β-d-glucopyranosidase, β-d-galactopyranosidase, β-d-xylopyranosidase, and α-l-arabinopyranosidase activity [22]. However, this enzyme showed a critical problem that the hydrolysis on PPD-type ginsenosides with α-l-arabinofuranoside was low or absent, and most of these ginsenosides were not hydrolyzed into C-K and accumulated. Among the PPD-type ginsnosides, Rc, compound Mc_1_ (C-Mc_1_) and compound Mc (C-Mc) contain α-l-arabinofuranoside in the dammarane skeleton. When the outer β-d-glucopyranoside at C-3 position in Rc is hydrolyzed, it is converted to C-Mc_1_, which is converted to C-Mc by cleaving the inner β-d-glucopyranoside in C-Mc_1_. β-Glycosidase from *S*. *solfataricus* does not hydrolyze α-l-arabinofuranoside linked to Rc, but slightly hydrolyzed α-l-arabinofuranoside linked to C-Mc because of its low α-l-arabinofuranosidase activity. As a result, most of it is accumulated. However, α-l-arabinofuranosidases from diverse microorganisms show no activity for other glycosides, and it is not suitable for C-K production [8]. Therefore, effective production of C-K from PPD-type ginsenosides requires a variant β-glucosidase with high hydrolysis activity for α-l-arabinofuranoside linked to ginsenosides.

In this study, C-Mc docking in homology model, sequence alignment with β-glycosidase from *Pyrococcus furiosus* containing α-l-arabinofuranosidase activity, and site-directed mutagenesis, time-course reactions were performed. As a result, a L213A variant with increased α-l-arabinofuranosidase activity was obtained. The activity of the L213A variant enzyme for α-l-arabinofuranoside linked to ginsenosides Rc, C-Mc_1_, and C-Mc was determined and compared to that of the wild-type enzyme. The conversion of ginsenoside Rc to C-K was significantly improved by using the L213A variant enzyme with increased *α*-l-arabinofuranosidase activity.

## Materials and methods

### Materials

Ginsenoside standards purchased from Ambo Institute (Seoul, Korea) and BTGin (Daejeon, Korea) were Rb_1_, Rc, F_2_, compound O (C-O), compound Y (C-Y), C-Mc, C-Mc_1_, C-K, Rb_2_, and Rd. Digoxin was purchased from Sigma (St. Louis. MO, USA) and was added to ginsenoside reaction solution as an internal standard for exact determination of ginsenosides in high-performance liquid chromatography (HPLC).

### Gene cloning and site-directed mutagenesis

β*-*Glycosidase gene of *S*. *solfataricus* DSM 1617 (DSMZ, Braunschweig, Germany) was cloned as previously described [23]. Site-directed mutagenesis was performed using a Muta-Direct Site Directed Mutagenesis kit (Intron, Seougnam, Korea), and it were made by polymerase chain reaction (PCR) using synthetic oligonucleotide primers. Primers used to replace the amino acid Leu213 with Ala213 are listed below: forward (5'-CATTCCACATTTAGTCTAGCTATTTTTGCTCCCATTTTTTGTGCATTATC GTGAAA-3'); reverse (5'-TTTCACGATAATGCACAAAAAATGGGAGCAAAAATAGCTAGACTAAA TGTGGAATG-3'). The sequence of mutated PCR product was confirmed by comparing with the DNA sequence of the β-glycosidase of *S*. *solfataricus* in the pET-24 vector. The PCR product was treated with Mutazyme enzyme for the digestion of the original DNA template. After enzyme treatment, the mutant gene was transformed into competent cells of *Escherichia coli*.

### Enzyme expression

*E*. *coli* expressing β-glycosidase from *S*. *solfataricus* was cultivated at 37°C with shaking at 200 rpm in a 2-L Erlenmeyer flask containing 450 mL of Luria-Bertani (LB) medium supplemented with 20 μg/mL of kanamycin. In order to induce enzyme expression, 0.1 mM of isopropyl-β-d-thiogalactopyranoside (IPTG) as a final concentration was added at 0.8 optical density at 600 nm of the culture broth. After induction, the culture temperature and agitation were reduced 16°C and 150 rpm, respectively, and cells were further incubated for 16 h.

### Enzyme preparation

Recombinant *E*. *coli* expressing wild-type or L213A variant enzyme was harvested and resuspended in 50 mM citrate/phosphate buffer at pH 5.5 or pH 4.5, respectively. The obtained cells were lysed using a sonicator (Sonic Dismembrator Model 100; Fisher Scientific, Pittsburgh, PA, USA) on ice for 20 min. Protein was acquired from the supernatant after centrifugation at 13,000×*g* for 10 min at 4°C, and it was heated at 70°C for 10 min to denature unwanted proteins derived from *E*. *coli* and to purify the thermophilic target protein. The suspension of heat-treated protein was centrifuged at 13,000×*g* for 10 min in order to remove aggregated proteins. The supernatant obtained was filtered using a 0.45 μm-sterile syringe filter, and the filtrate was used as the purified enzyme in subsequent experiments.

### Arabinofuranosidase activity

Reactions were conducted at 80°C in 50 mM citrate/phosphate buffer (pH 5.5) containing 0.4 mg/mL ginsenoside Rc or C-Mc, and 0.15 mg/mL wild-type or variant enzyme. α-l-Arabinofuranosidase activities of the wild-type and variant enzymes were determined on the produced concentrations of ginsenoside Rd or C-K.

### Substrate specificity

The substrate specificities of the wild-type and L213A variant β-glycosidases from *S*. *solfataricus* were determined by measuring the specific activities for ginsenosides Rb_1_, Rb_2_, Rd, F_2_, C-O, C-Y, C-Mc_1_, and C-Mc. The specific activity of the variant enzyme for ginsenoside Rc was not determined because it converted Rc to not only C-Mc_1_ but also Rd. The reactions of the wild-type and L213A variant β-glycosidases from *S*. *solfataricus* were carried out at 95°C in 50 mM citrate/phosphate buffer at pH 5.5 and pH 4.5, respectively, and 4% (v/v) dimethyl sulfoxide (DMSO) by varying the enzyme concentration from 0.01 to 0.5 mg/mL and the time from 5 to 30 min. The specific activity was defined as the decreased amount of the substrate ginsenoside (nmol) per min per mg of protein.

### Ligand docking

β-Glycosidase from *S*. *solfataricus* was modeled using the Build Homology Models module in Discovery Studio 4.0 (Accerlys, San Diego, CA, USA) based on the determined structure of β-glycosidase from *S*. *solfataricus* (Protein Data Bank [PDB] entry, 2CEQ) as a template. The substrate C-Mc was docked into the active-site pocket in the models of *S*. *solfataricus* β-glycosidase using C-DOCKER, because C-Mc has a similar structure to Rc at C-20 position. The active site defined as a sphere of radius 10 Å around the substrate-binding pocket. The receptor-ligand (enzyme-substrate) docking models giving the lowest interaction energy were chosen for subsequent rounds of docking. Changes in the binding energy between receptor and ligand (*∆E*_Binding_) were defined as *E*_Complex_ − *E*_Ligand_ − *E*_Receptor_.

### Production of C-K from ginsenoside Rc

The reactions of the wild-type and L213A variant β-glycosidases from *S*. *solfataricus* for C-K production using ginsenoside Rc as a substrate were performed in 50 mM citrate/phosphate buffer at pH 5.5 and pH 4.5, respectively, containing 4 mM ginsenoside Rc and 12 mg/mL enzyme. The temperature in these reactions was reduced to 85°C because of the thermostability of this enzyme. The half-life at this temperature was 30 h, indicating that the enzyme is stable during the reactions. The reaction mixtures were sampled, and ginsenoside concentrations were determined using HPLC.

### Analytical methods

Digoxin as an internal standard and *n*-butanol with an equal volume were added to the reaction solution. The solvent in the mixed solution was evaporated, and methanol was added to the evaporated sample. The concentrations of ginsenosides were determined using an HPLC system (Agilent 1100, Agilent, Santa Clara, CA, USA) at a wavelength of 203 nm with a C18 column (YMC, Kyoto, Japan). The column was eluted at 37°C with a linear gradient of acetonitrile/water ranging from 30:70 to 90:10 (v/v) for 95 min at a flow rate of 1 mL/min. Ginsenoside type was identified using the same retention time as that for the ginsenoside standards.

## Results and discussion

### Selection of a C-K-producing enzyme from ginsenoside Rc

The enzymatic hydrolysis of ginsenoside Rc to PPD-type minor ginsenosides by various microbial enzymes, including α-l-arabinofuranosidases, β-glucosidases, and β-glycosidases is present in Table 1. α-l-Arabinofuranosidases from various microorganisms convert Rc to Rd by hydrolyzing only α-l-arabinofuranoside, while the Rd is not further hydrolyzed by these enzymes. β-Glucosidases and β-glycosidases from various microorganisms transform ginsenoside Rc to C-Mc_1_, C-Mc, aglycone protopanaxadiol (APPD), and C-K. Among these enzymes, only two enzymes could convert ginsenoside Rc to C-K. β-Glycosidase from *P*. *furiosus* produced C-K as an intermediate, which was completely hydrolyzed to APPD [24], and β-glycosidase from *S*. *solfataricus* produced C-K as an end product, which hydrolyzes two glucose in C-3, but does not hydrolyze arabinose in C-20 at Rc. Thus, β-glycosidase from *P*. *furiosus* and *S*. *solfataricus* were selected as a comparison target enzyme and a source enzyme for the increased biotransformation of ginsenoside Rc to C-K, respectively.

**Table 1:** Enzymatic hydrolysis of ginsenoside Rc to PPD-type ginsenosides.

Microorganism	Enzyme	Intermediate product	Product	Reference
*Thermotoga thermarum*	α-l-Arabinofuranosidase	NR	Rd	[30]
*Leuconostoc sp*.	α-l-Arabinofuranosidase	NR	Rd	[31]
*Caldicellulosiruptor saccharolyticus*	α-l-Arabinofuranosidase	NR	Rd	[32]
*Rhodanobacter ginsenosidimutans*	α-l-Arabinofuranosidase	NR	Rd	[28]
*Bifidobacterium longum*	α-l-Arabinofuranosidase	NR	Rd	[33]
*Bifidobacterium breve*	α-l-Arabinofuranosidase	NR	Rd	[34]
*Panax ginseng*	α-l-Arabinofuranosidase	NR	Rd	[35]
*Thermus caldophilus*	β-Glycosidase	NR	Rd	[36]
*Sphingopyxis alaskensis*	β-Glucosidase	NR	C-Mc_1_	[37]
*Arthrobacter chlorophenolicus*	β-Glucosidase	NR	C-Mc_1_	[38]
*Sphingomonas* sp. 2F2	β-Glucosidase	NR	C-Mc_1_	[39]
*Penicillium aculeatum*	β-Glucosidase	C-Mc_1_	C-Mc	[40]
*Dictyoglomus turgidum*	β-Glucosidase	ND	C-Mc	[41]
*Sulfolobus acidocaldarius*	β-Glycosidase	ND	C-Mc	[42]
*Pyrococcus furiosus*	β-Glycosidase	Rd, C-K	APPD	[24]
*Sulfolobus solfataricus*	β-Glycosidase	C-Mc	C-K	[29]

NR, not reported; ND, not detected

β-Glycosidase from *S*. *solfataricus* is an effective C-K producer because the enzyme can hydrolyze β-d-glucopyranose linked to C-3, outer β-d-glucopyranose, α-l-arabinopyranose, α-l-arabinofuranose, and β-d-xylopyranose linked to C-20 of the dammarane skeleton in PPD-type ginsenosides but does not hydrolyze inner β-d-glucopyranose linked to C-20 [25–28]. β-Glycosidase from *S*. *solfataricus* can hydrolyze β-d-glucopyranoside linked to ginsenosides such as Rc, C-Mc_1_, and C-Mc, which are converted into C-Mc1, C-Mc, and C-K, respectively. However, this enzyme showed low or no hydrolytic activity on α-l-arabinofuranoside linked to Rc, C-Mc_1_, and C-Mc. For the efficient biotransformation of ginsenoside Rc to C-K, a β-glycosidase variant with increased α-l-arabinofuranosidase activity should be achieved.

### Achievement of a variant β-glycosidase from *S*. *solfataricus* with increased α-l-arabinofuranosidase activity

**Fig 1:**
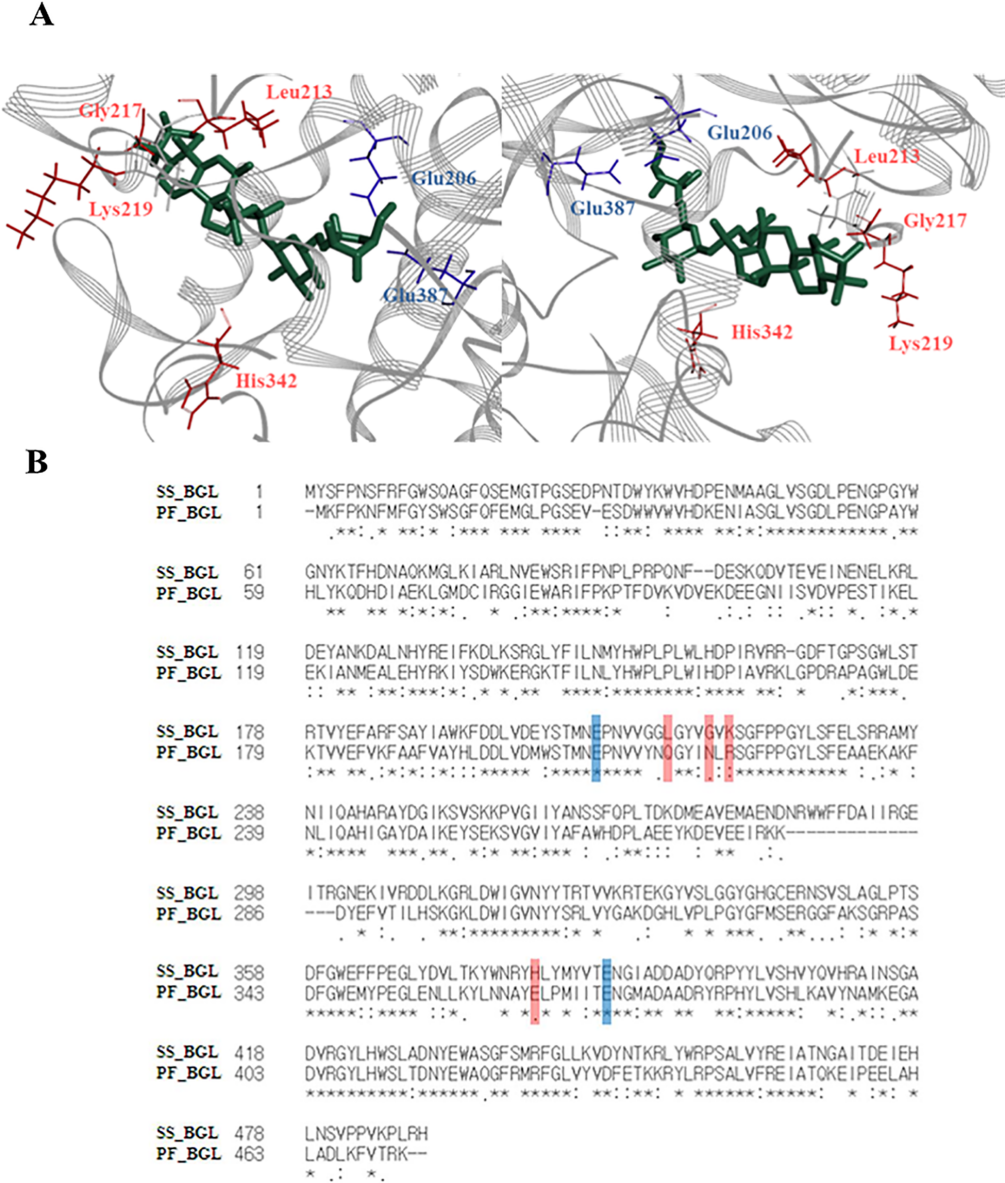
Construction of ligand docked pose and sequence alignment on *S*. *solfataricus* β*-*glycosidase. (A) Docking of C-Mc to *S*. *solfataricus* β*-*glycosidase in the homology model. The C-Mc ligand was docked to the active site of β*-*glycosidase from *S*. *solfataricus* (2CEQ), and amino acid residues within 4 Å distance of the substrate in the ligand docked model were selected. The selected non-catalytic residues, catalytic residues, and C-Mc are colored with red, blue, and green, respectively. (B) Amino acid sequence alignment of *S*. *solfataricus* β*-*glycosidase with *P*. *furiosus* β*-*glycosidase. The catalytic residues (Glu206 and Glu387) and non-catalytic residues (Leu213, Glu217, Lys219, and His342) of *S*. *solfataricus* β*-*glycosidase are colored with red and blue boxes, respectively.

In order to identify the residue involved in α-l-arabinofuranoside hydrolysis, a C-Mc docked model was constructed based on the reported crystal structure of β-glycosidase from *S*. *solfataricus*. Rc could not docked to the wild-type β-glycosidase because it has a large molecular size. The outer α-l-arabinofuranoside at C-20 of C-Mc is the same as that of the smaller molecular size Rc. Therefore, we used C-Mc in docking to β-glycosidase from *S*. *solfataricus*, instead of Rc. In the ligand docked model, four non-catalytic and two catalytic residues within a 4 Å of the substrate were identified. The non-catalytic residues were Leu213, Glu217, Lys219, and His342 and the catalytic residues were Glu206 and Glu387 (Fig 1A). Since β-glycosidase from *P*. *furiosus* showed high hydrolytic activity on α-l-arabinofuranoside linked to ginsenosides Rc and C-Mc [24], the sequence of β-glycosidase from *S*. *solfataricus* was aligned with that of β-glycosidase from *P*. *furiosus* that has α-l-arabinofuranosidase activity to determine candidate residues involved in α-l-arabinofuranosidase activity in β-glycosidase from *S*. *solfataricus* (Fig 1B). Glu206, and Glu387 were all conserved in the two enzymes. Therefore, Leu213, Glu217, Lys219, and His342 were selected as candidate residues.

The candidate residues were mutated as shown in Table 2. The wild-type and Lys219, and His342 variant enzymes showed no hydrolytic activity on α-l-arabinofuranoside linked to Rc, while the Glu217 variant enzyme exhibited no hydrolytic activity on α-l-arabinofuranoside linked to C-Mc. However, the L213A, L213Q, and L213W variant enzymes hydrolyzed α-l-arabinofuranoside linked to both Rc and C-Mc, while the L213A variant enzyme showed higher α-l-arabinofuranosidase activity than those of the L213Q and L213W variant enzymes. Thus, we used the L213A variant enzyme for hydrolysis of α-l-arabinofuranoside linked to Rc and C-Mc. α-l-Arabinofuranosidase hydrolyzes α-l-arabinofuranoside linked to Rc to form Rd, but not β-d-glucopyranosides linked to Rd and the wild-type β-glycosidase from *S*. *solfataricus* cannot hydrolyzes α-l-arabinofuranoside linked to Rc, indicating that the enzymes do not convert Rd and Rc into C-K, respectively. In contrast, the L213A variant can convert Rd and Rc into C-K.

**Table 2:** Produced concentrations of ginsenoside Rd and C-K from ginsenoside Rc and C-Mc, respectively, by the wild-type and variant β-glycosidases of *S*. *solfataricus*. Numerical values in round brackets present the experimental data under optimum conditions (95°C, pH 4.5, and 4% DMSO).

Enzyme	Product (*μ*M)	
	Rd	C-K
Wild-type	0	198 ± 3.0
L213A	63.4 ± 1.1 (75.7±0.8)	386 ± 2.0 (437 ± 3.0)
L213Q	19.7 ± 0.4	343 ± 4.0
L213R	0	0
L213W	11.9 ± 0.2	209 ± 4.0
L213S	10.3 ± 0.1	0
L213G	26.1 ± 0.5	0
L213D	2.7 ± 0.1	0
L213E	1.4± 0.1	0
L213H	1.8 ± 0.1	0
G217A	4.8 ± 0.1	0
G217N	23.4 ± 0.4	0
K219R	0	259 ± 2.0
H342A	0	243.0 ± 3.0
H342E	0	0

ND, not detected

### Substrate specificities of the wild-type and L213A variant β-glycosidases from *S*. *solfataricus* for PPD-type ginsenosides

The substrate specificities of the wild-type and L213A variant β-glycosidases from *S*. *solfataricus* were determined using the PPD-type ginsenosides C-Mc, C-Mc_1_, Rd, Rb_1_, Rb_2_, F_2_, C-O, and C-Y (Table 3). The specific hydrolytic activities for the wild-type and L213A variant enzymes followed the order F2 > Rb_1_ > C-O > C-Y > Rb_2_ > Rd > C-Mc> C-Mc_1_ and F_2_ > Rb_1_ > C-O > C-Y > Rb_2_ > C-Mc > Rd > C-Mc_1_, respectively. The wild-type enzyme exhibited no α-l-arabinofuranosidase activity for ginsenoside Rc, but the L213A variant enzyme showed. The activity of the L213A variant enzyme for C-Mc was 2.2-fold higher than that of the wild-type enzyme, respectively, whereas that for C-Y was 1.5-fold lower, indicating increased α-l-arabinofuranosidase activity and decreased α-l-arabinopyranosidase activity, respectively. To explain the increased α-l-arabinofuranosidase activity of the L213A variant, we calculated the changes in binding energy (*∆E*_binding_) of the ligand-binding enzymes. The changes in the binding energy of the wild-type and L213A variant β-glycosidases docked with C-Mc were −86.5 and −177.2 kcal mol^−1^, respectively. The 2-fold lower binding energy of the variant β-glycosidase suggests that it has a higher regioselectivity and forms a more stable complex.

**Table 3:** Substrate specificity of the wild-type and L213A variant β- glycosidase from *S*. *solfataricus* for the PPD-type ginsenosides.

Substrate	Product	Specific activity (nmol/min/mg)	
		Wild-type	L213A
C-Mc	C-K	66.1 ± 0.9	146 ± 0.5
C-Mc_1_	C-K	12.6 ± 1.3	56.7 ± 0.9
Rd	C-K	211 ± 2.9	80.2 ± 1.8
Rb_1_	C-K	8560 ± 120	13650 ± 290
Rb_2_	C-K	520 ± 10	540 ± 10
F_2_	C-K	24100 ± 150	33400 ± 210
C-O	C-K	4240 ± 110	4050 ± 20
C-Y	C-K	1740 ± 10	1180 ± 10

### Transformation pathways of the wild-type and L213A variant β-glycosidases from *S*. *solfataricus*

**Fig 2:**
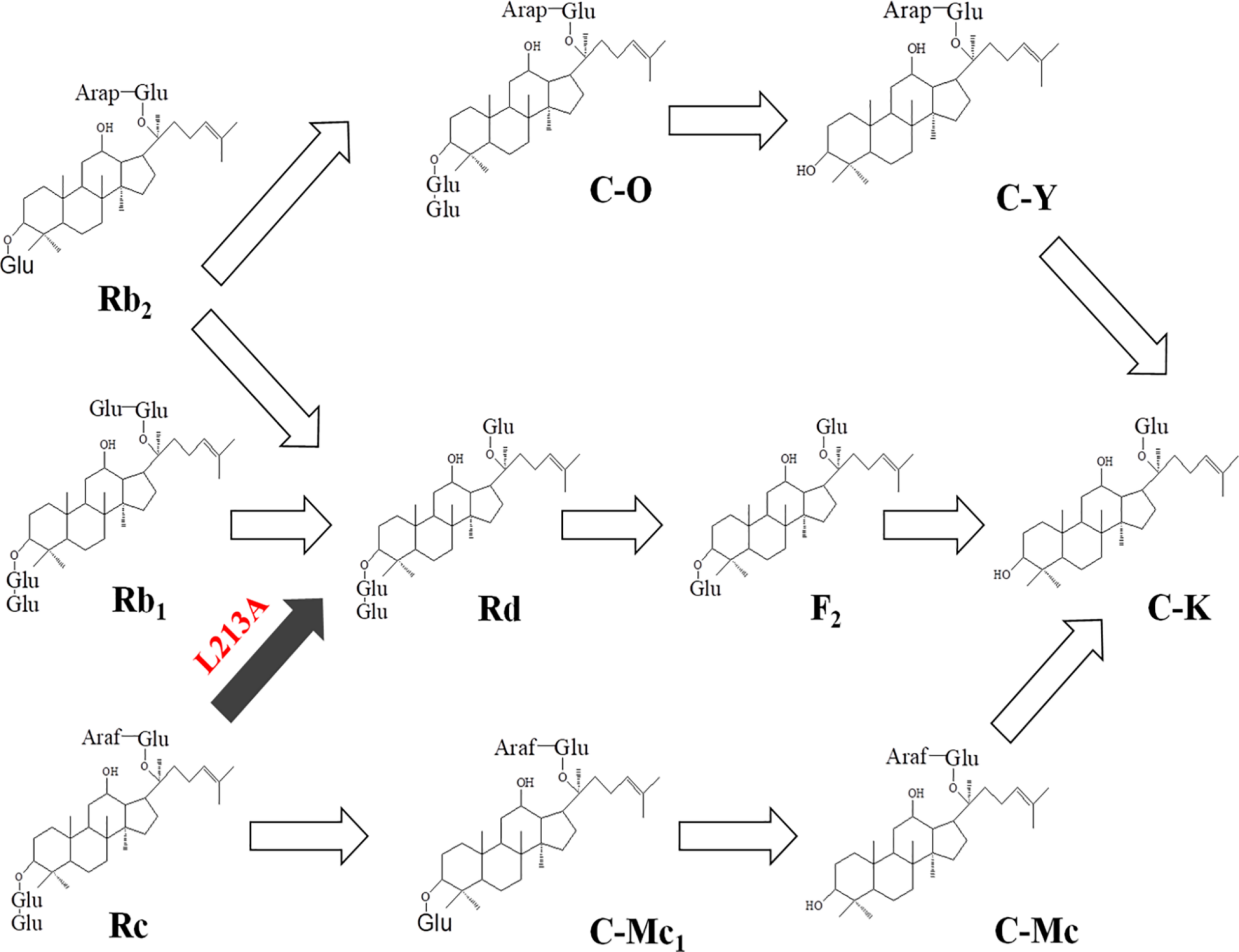
Biotransformation pathways from ginsenosides Rb_1_, Rb_2_, and Rc to C-K by the wild-type and L213A variant β-glycosidases from *S*. *solfataricus*. The black arrow indicates newly formed hydrolytic activity by the L213A variant.

The transformation pathway of ginsenoside Rb_1_, Rb_2_, Rc, or Rd to C-K by the wild-type β-glycosidase from *S*. *solfataricus* was previously reported as Rb_1_ or Rb_2_ → Rd → F_2_ → C-K, and Rc → C-Mc_1_ → C-Mc → C-K [29]. Ginsenoside Rc was not converted to Rd due to an absence of α-l-arabinofuranosidase activity of the wild-type enzyme for ginsenoside Rc. The L213A variant enzyme included the pathway of Rc → Rd → F_2_ → C-K in addition to the previous one due to its α-l-arabinofuranosidase activity for ginsenoside Rc (Fig 2). The wild-type and L213A variant enzymes converted C-O and C-Y into C-K (Table 3). The enzymes converted Rb2 into not only Rd as a major product but also C-O as a minor product. Thus, we established another C-K-producing transformation pathway: Rb_2_ → C-O → C-Y → C-K in the wild-type and L213A variant enzymes.

### C-K production from ginsenoside Rc and C-Mc by wild-type and L213A variant β-glycosidases from *S*. *solfataricus*

**Fig 3:**
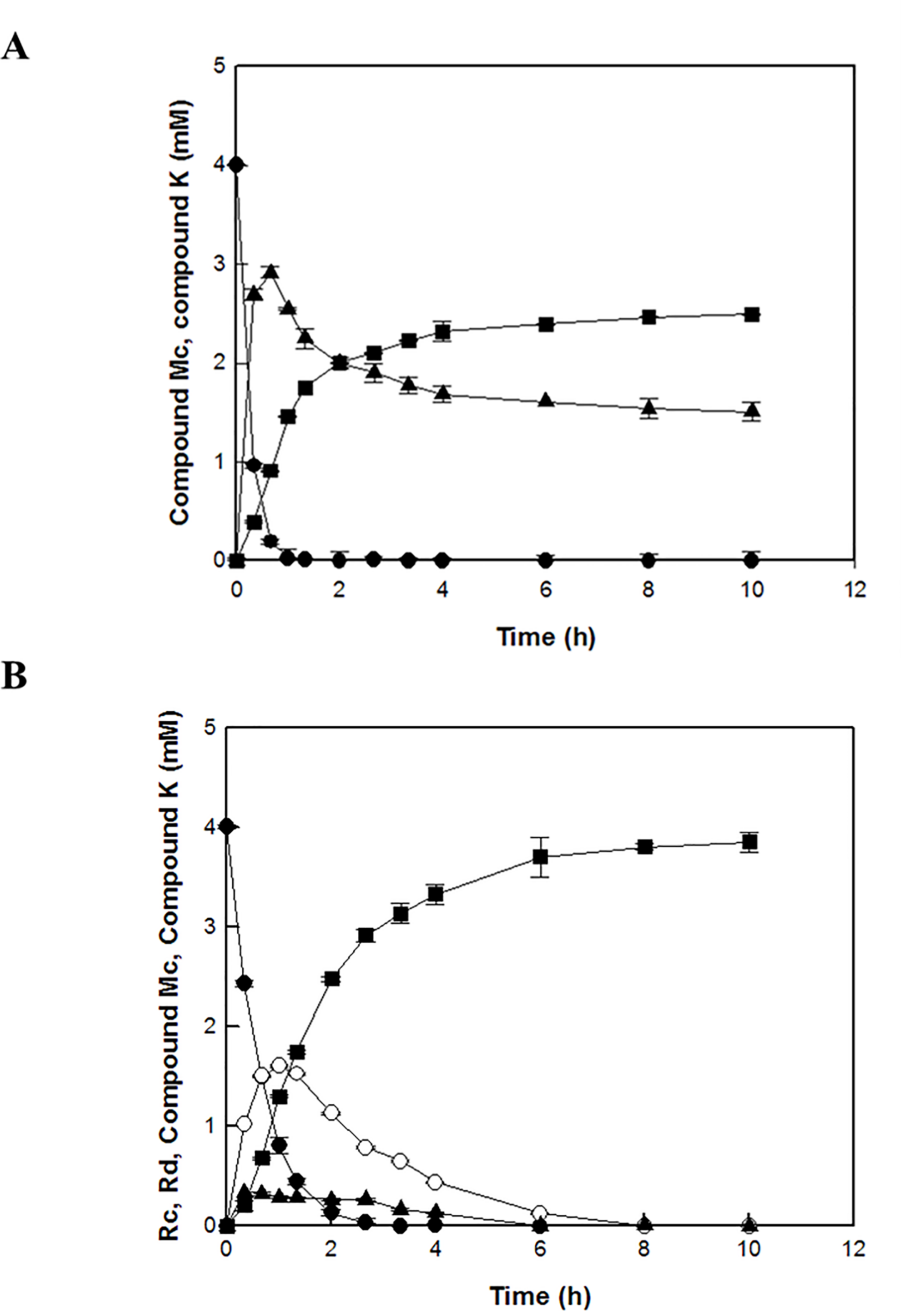
**C-K production from ginsenoside Rc via C-Mc with Rd and C-Mc as intermediates by (A) the wild-type and (B) L213A variant *β-*glycosidases from *S*. *solfataricus*, respectively.** Ginsenoside Rc (filled circle), Rd (open circle), C-Mc (filled triangle), and C-K (filled square).

**Fig 4:**
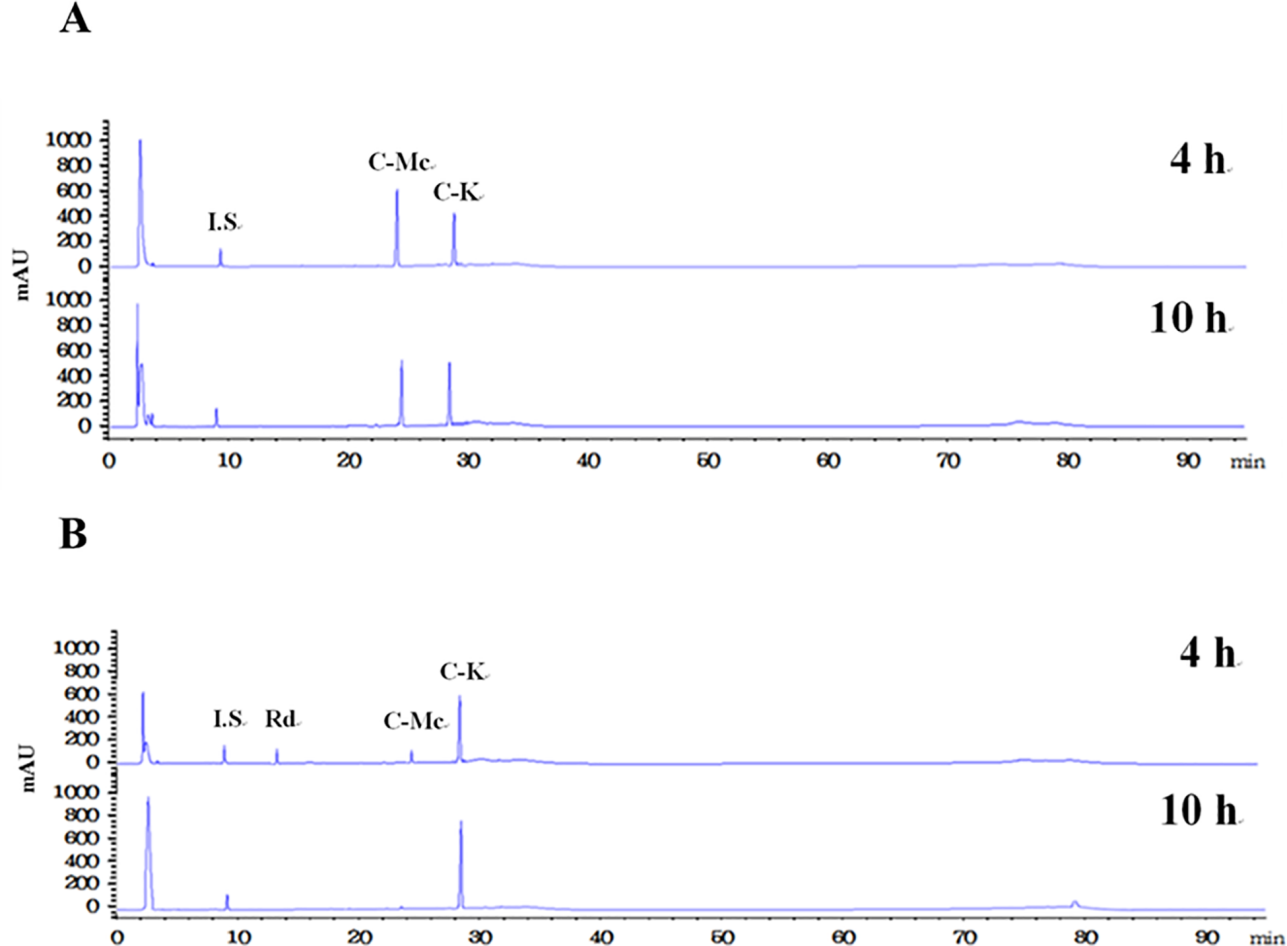
HPLC profiles of the reaction solutions obtained after 4 h and 10 h for the production of C-K from ginsenoside Rc by (A) the wild-type and (B) L213A variant β*-*glycosidases from *S*. *solfataricus*.

**Fig 5:**
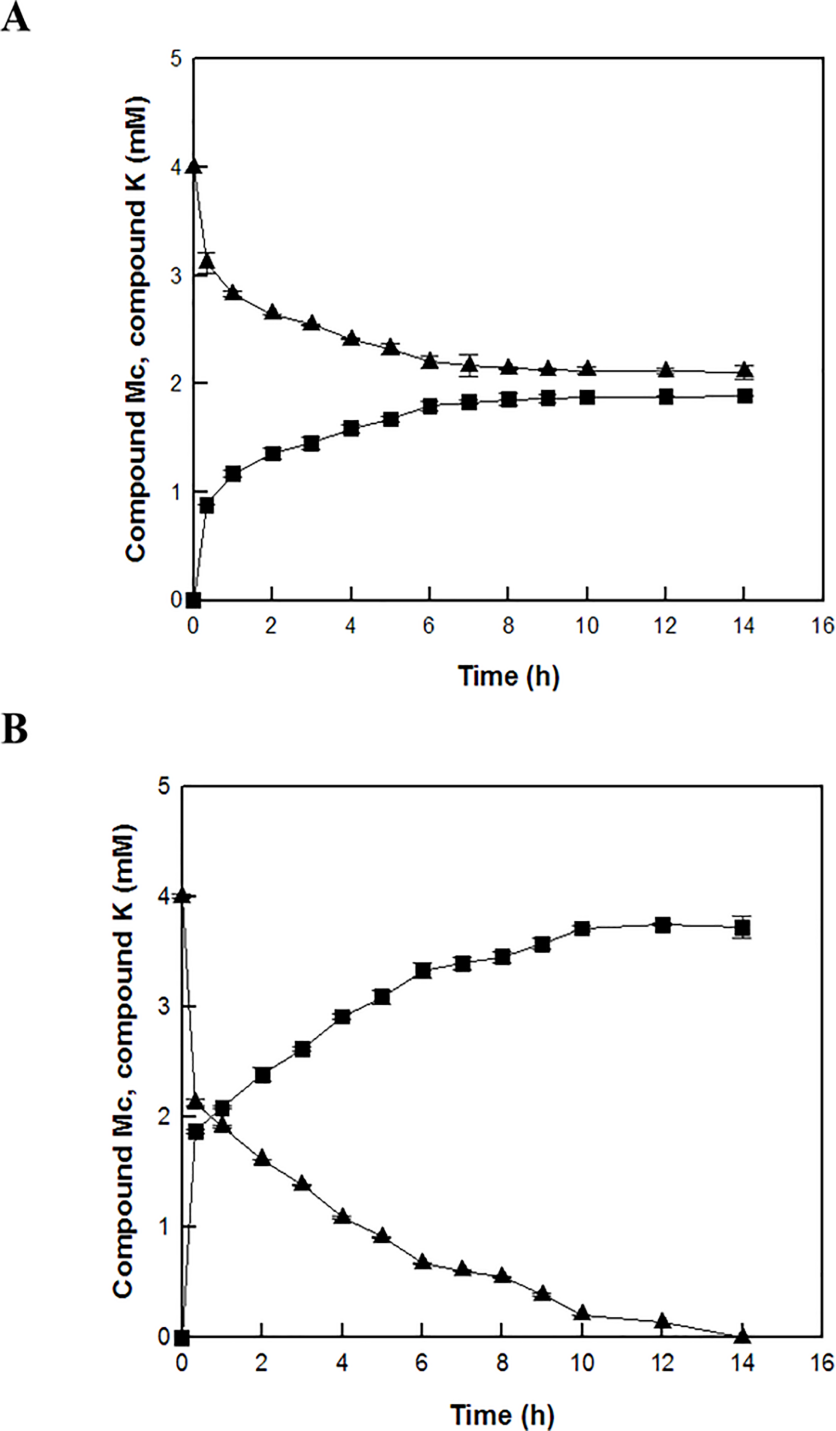
**C-K production from ginsenoside Mc by (A) the wild-type and (B) L213A variant *β-*glycosidases from *S*. *solfataricus*.** Ginsenoside C-Mc (filled triangle), and C-K (filled square).

**Fig 6:**
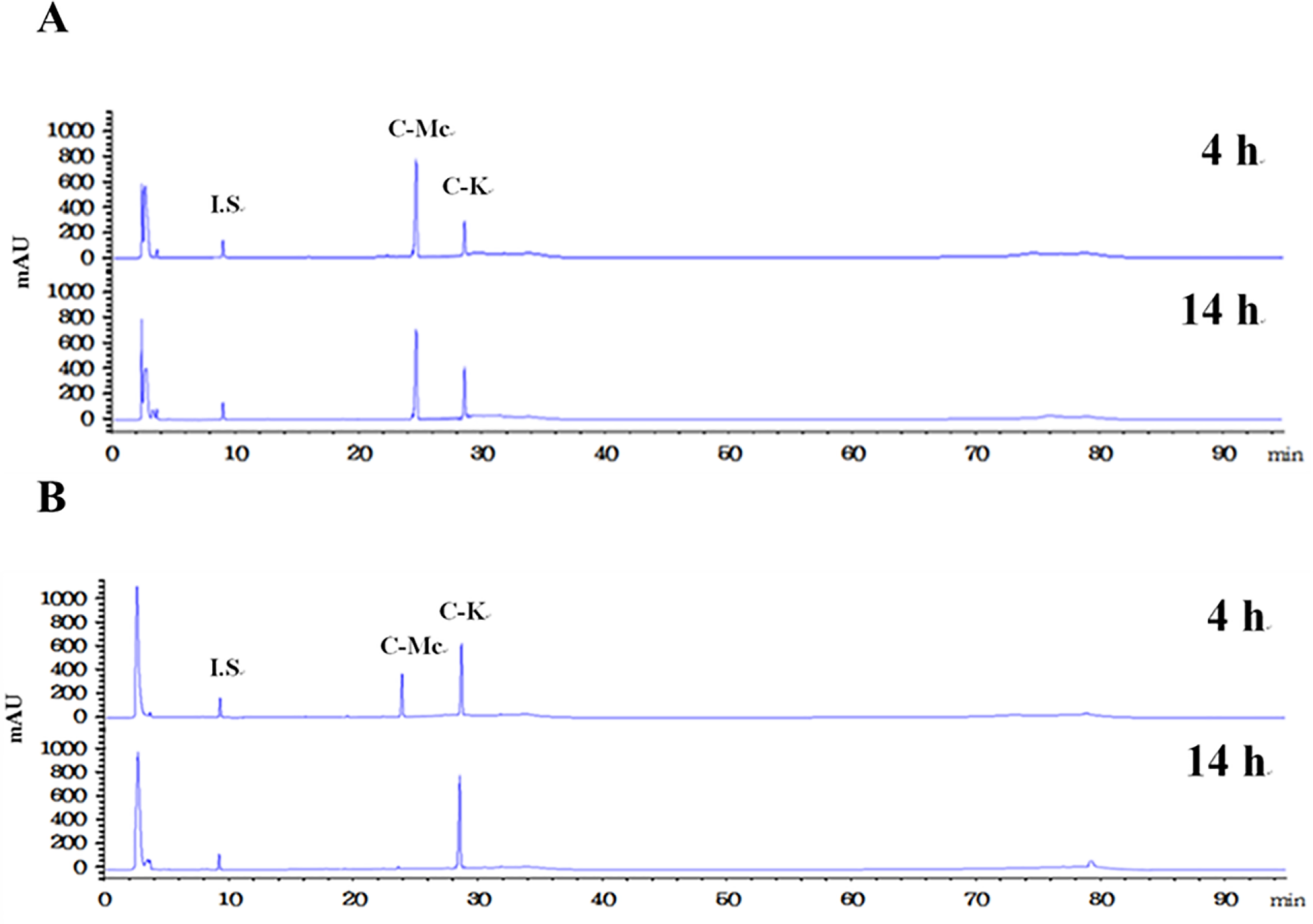
HPLC profiles of the reaction solutions obtained after 14 h for the production of C-K from ginsenoside Mc by (A) the wild-type and (B) L213A variant enzymes from *S*. *solfataricus*.

The time-course reactions of Rc to C-K by the wild-type and L213A variant enzymes were quantitatively analyzed by the HPLC system. The wild-type and L213A variant β-glycosidases from *S*. *solfataricus* converted 4 mM ginsenoside Rc to 2.5 mM C-K via C-Mc and 3.8 mM C-K via Rd and C-Mc, respectively, in 10 h. The molar conversions of these reactions were 62% and 97%, respectively (Fig 3 and Fig 4). The amount of C-K produced from Rc by the variant enzyme was 1.5-fold higher than that by the wild-type enzyme. The wild-type and variant enzymes converted 4 mM C-Mc to 1.9 mM and 3.8 mM C-K, respectively, in 14 h. The molar conversions were 47% and 97%, respectively (Fig 5 and Fig 6). The amount of C-K produced from Rc by the variant enzyme was 2.0-fold higher than that by the wild-type enzyme.

## Conclusions

β-glycosidase from *S*. *solfataricus* is the most efficient producer of C-K from PPD-type ginsenosides. However, this enzyme has a critical problem in that low or no hydrolytic activity is seen on α-l-arabinofuranoside linked to ginsenosides. To solve this problem, an L213A variant with increased α-l-arabinofuranosidase activity was obtained through protein engineering based on a ligand docked homology model and sequence alignment. The variant enzyme converted ginsenoside Rc and C-Mc to C-K with both molar conversions of 97%, which was 35% and 50% higher than the wild-type enzyme, respectively. Therefore, protein engineering is a useful tool for enhancing the hydrolytic activity of specific glycosides linked to ginsenosides.

## References

[1] Xiang YZ, Shang HC, Gao XM, Zhang BL. A comparison of the ancient use of ginseng in traditional Chinese medicine with modern pharmacological experiments and clinical trials. Phytother Res. 2008;22(7):851–8. doi: 10.1002/ptr.2384 . 18567057 10.1002/ptr.2384

[2] Lu JM, Yao Q, Chen C. Ginseng compounds: an update on their molecular mechanisms and medical applications. Curr Vasc Pharmacol. 2009;7(3):293–302. https://doi.org/PMC2928028. . 19601854 10.2174/157016109788340767 PMC2928028

[3] Yoshikawa M, Morikawa T, Kashima Y, Ninomiya K, Matsuda H. Structures of new dammarane-type triterpene saponins from the flower buds of *Panax notoginseng* and hepatoprotective effects of principal ginseng saponins 1. J Nat Prod. 2003;66(7):922–7. https://doi.org/10.1021/np030015l. . 12880307 10.1021/np030015l

[4] Yayeh T, Yun K, Jang S, Oh S. Morphine dependence is attenuated by red ginseng extract and ginsenosides Rh2, Rg3, and compound K. J Ginseng Res. 2016;40(4):445–52. https://doi.org/10.1016/j.jgr.2016.08.006. . 27746699 10.1016/j.jgr.2016.08.006 PMC5052441

[5] Im KT, Kim JS, Min HY. Ginseng, the natural effectual antiviral: Protective effects of Korean Red Ginseng against viral infection. J Ginseng Res. 2015;40:309–14. https://doi.org/10.1016/j.jgr.2015.09.002. . 27746682 10.1016/j.jgr.2015.09.002 PMC5052424

[6] Joh EH, Lee IA, Jung IH, Kim DH. Ginsenoside Rb1 and its metabolite compound K inhibit IRAK-1 activation—the key step of inflammation. Biochem Pharmacol 2011;82(3):278–86. https://doi.org/10.1016/j.bcp.2011.05.003. . 21600888 10.1016/j.bcp.2011.05.003

[7] Wu CF, Bi XL, Yang JY, Zhan JY, Dong YX, Wang JH, et al Differential effects of ginsenosides on NO and TNF-α production by LPS-activated N9 microglia. Intl Immunopharmacol. 2007;7(3):313–20. https://doi.org/10.1016/j.intimp.2006.04.021. . 17276889 10.1016/j.intimp.2006.04.021

[8] Shin KC, Oh DK. Classification of glycosidases that hydrolyze the specific positions and types of sugar moieties in ginsenosides. Crit Rev Biotechnol. 2016;36(6):1036–49. https://doi.org/10.3109/07388551.2015.1083942 . 26383974 10.3109/07388551.2015.1083942

[9] Kim MK, Lee JW, Lee KY, Yang D. Microbial conversion of major ginsenoside Rb1 to pharmaceutically active minor ginsenoside Rd. J Microbiol. 2005;43(5):456–62. . 16273039

[10] Liu KK, Wang QT, Yang SM, Chen JY, Wu HX, Wei W. Ginsenoside compound K suppresses the abnormal activation of T lymphocytes in mice with collagen-induced arthritis. Acta Pharmacol Sin. 2014;35(5):599–612. https://doi.org/10.1038/aps.2014.7. . 24727939 10.1038/aps.2014.7 PMC4814039

[11] Law CM, Kwok H, Poon PY, Lau CC, Jiang ZH, Tai WS, et al Ginsenoside compound K induces apoptosis in nasopharyngeal carcinoma cells via activation of apoptosis-inducing factor. Chin Med. 2014;9(1):11 https://doi.org/10.1186/1749-8546-9-11. . 24690317 10.1186/1749-8546-9-11 PMC4021625

[12] Zhou W, Feng MQ, Li JY, Zhou P. Studies on the preparation, crystal structure and bioactivity of ginsenoside compound K. J Asian Nat Prod Res. 2006;8:519–27. https://doi.org/10.1080/10286020500208600. . 16931427 10.1080/10286020500208600

[13] Chen Y, Xu Y, Zhu Y, Li X. Anti-cancer effects of ginsenoside compound k on pediatric acute myeloid leukemia cells. Cancer Cell Int. 2013;13(1):24 https://doi.org/10.1186/1475-2867-13-24. . 23497352 10.1186/1475-2867-13-24 PMC3602037

[14] Hu C, Song G, Zhang B, Liu Z, Chen R, Zhang H, et al Intestinal metabolite compound K of panaxoside inhibits the growth of gastric carcinoma by augmenting apoptosis via Bid-mediated mitochondrial pathway. J Cell Mol Med. 2012;16(1):96–106. https://doi.org/10.1111/j.1582-4934.2011.01278.x. . 21323864 10.1111/j.1582-4934.2011.01278.x PMC3823096

[15] Choo MK, Park EK, Han MJ, Kim DH. Antiallergic activity of ginseng and its ginsenosides. Planta Med. 2003;69(06):518–22. https://doi.org/10.1055/s-2003-40653. . 12865969 10.1055/s-2003-40653

[16] Yuan HD, Kim SJ, Chung SH. Beneficial effects of IH-901 on glucose and lipid metabolisms via activating adenosine monophosphate–activated protein kinase and phosphatidylinositol-3 kinase pathways. Metabolism. 2011;60(1):43–51. https://doi.org/10.1016/j.metabol.2009.12.024. . 20153001 10.1016/j.metabol.2009.12.024

[17] Shin DJ, Kim JE, Lim TG, Jeong EH, Park G, Kang NJ, et al 20-*O*-β-D-Glucopyranosyl-20(S)-protopanaxadiol suppresses UV-induced MMP-1 expression through AMPK-mediated mTOR inhibition as a downstream of the PKA-LKB1 pathway. J Cell Biochem 2014;115(10):1702–11. https://doi.org/10.1002/jcb.24833 . 24821673 10.1002/jcb.24833

[18] Kimura Y, Sumiyoshi M, Kawahira K, Sakanaka M. Effects of ginseng saponins isolated from red ginseng roots on burn wound healing in mice. Brit J Pharmacol. 2006;148(6):860–70. https://doi.org/10.1038/sj.bjp.0706794. . 16770323 10.1038/sj.bjp.0706794 PMC1617068

[19] Kim SJ, Kang BY, Cho SY, Sung DS, Chang HK, Yeom MH, et al Compound K induces expression of hyaluronan synthase 2 gene in transformed human keratinocytes and increases hyaluronan in hairless mouse skin. Biochem Biophys Res Commun 2004;316(2):348–55. https://doi.org/10.1016/j.bbrc.2004.02.046. . 15020224 10.1016/j.bbrc.2004.02.046

[20] He D, Sun J, Zhu X, Nian S, Liu J. Compound K increases type I procollagen level and decreases matrix metalloproteinase-1 activity and level in ultraviolet-A-irradiated fibroblasts. J Formos Med Assoc. 2011;110(3):153–60. doi: 10.1016/S0929-6646(11)60025-9 . 21497278 10.1016/S0929-6646(11)60025-9

[21] Park CS, Yoo MH, Noh KH, Oh DK. Biotransformation of ginsenosides by hydrolyzing the sugar moieties of ginsenosides using microbial glycosidases. Appl Microbiol Biotechnol. 2010;87(1):9–19. https://doi.org/10.1007/s00253-010-2567-6 . 20376631 10.1007/s00253-010-2567-6

[22] Nucci R, Moracci M, Vaccaro C, Vespa N, Rossi M. Exo-glucosidase activity and substrate specificity of the beta-glycosidase isolated from the extreme thermophile *Sulfolobus solfataricus*. Biotechnol Appl Biochem. 1993;17(2):239–50. https://doi.org/10.1111/j.1470-8744.1993.tb00242.x. . 8484908

[23] Shin KC, Choi HY, Seo MJ, Oh DK. Compound K production from red ginseng extract by β-glycosidase from *Sulfolobus solfataricus* supplemented with α-L-arabinofuranosidase from *Caldicellulosiruptor saccharolyticus*. PLoS One. 2015;10(12):e0145876 https://doi.org/10.1371/journal.pone.0145876. . 26710074 10.1371/journal.pone.0145876 PMC4692446

[24] Yoo MH, Yeom S, Park CS, Lee KW, Oh DK. Production of aglycon protopanaxadiol via compound K by a thermostable β-glycosidase from *Pyrococcus furiosus*. Appl Microbiol Biotechnol. 2011;89(4):1019–28. https://doi.org/10.1007/s00253-010-2960-1. . 21052989 10.1007/s00253-010-2960-1

[25] Yan Q, Zhou W, Shi XL, Zhou P, Ju DW, Feng MQ. Biotransformation pathways of ginsenoside Rb1 to compound K by β-glucosidases in fungus *Paecilomyces Bainier* sp 229. Process Biochem. 2010;45(9):1550–6. https://doi.org/10.1016/j.procbio.2010.06.007. PMID: WOS:000281091700016.

[26] Kim SH, Min JW, Quan LH, Lee S, Yang DU, Yang DC. Enzymatic Transformation of Ginsenoside Rb1 by *Lactobacillus pentosus* Strain 6105 from Kimchi. J Ginseng Res. 2012;36(3):291–7. https://doi.org/10.5142/jgr.2012.36.3.291. . 23717130 10.5142/jgr.2012.36.3.291 PMC3659591

[27] Quan LH, Piao JY, Min JW, Yang DU, Lee HN, Yang DC. Bioconversion of Ginsenoside Rb1 into Compound K by *Leuconostoc Citreum* Lh1 Isolated from Kimchi. Braz J Microbiol. 2011;42(3):1227–37. https://doi.org/10.1590/S1517-838220110003000049. PMID: WOS:000297756800049. 24031746 10.1590/S1517-838220110003000049 PMC3768781

[28] Quan LH, Jin Y, Wang C, Min JW, Kim YJ, Yang DC. Enzymatic transformation of the major ginsenoside Rb2 to minor compound Y and compound K by a ginsenoside-hydrolyzing β-glycosidase from *Microbacterium esteraromaticum*. J Ind Microbiol Biot. 2012;39(10):1557–62. https://doi.org/10.1007/s10295-012-1158-1. . 22717707 10.1007/s10295-012-1158-1

[29] Noh KH, Son JW, Kim HJ, Oh DK. Ginsenoside compound K production from ginseng root extract by a thermostable β-glycosidase from *Sulfolobus solfataricus*. Biosci Biotech Biochem. 2009;73(2):316–21. https://doi.org/10.1271/bbb.80525 . 19202288 10.1271/bbb.80525

[30] Xie J, Zhao D, Zhao L, Pei J, Xiao W, Ding G, et al Characterization of a novel arabinose-tolerant α-L-arabinofuranosidase with high ginsenoside Rc to ginsenoside Rd bioconversion productivity. J Appl Microbiol. 2016;120(3):647–60. https://doi.org/10.1111/jam.13040. . 26725313 10.1111/jam.13040

[31] Liu QM, Jung HM, Cui CH, Sung BH, Kim JK, Kim SG, et al Bioconversion of ginsenoside Rc into Rd by a novel α-L-arabinofuranosidase, Abf22-3 from *Leuconostoc* sp. 22–3: cloning, expression, and enzyme characterization. Antonie Van Leeuwenhoek. 2013;103(4):747–54. https://doi.org/10.1007/s10482-012-9856-2. . 23224374 10.1007/s10482-012-9856-2

[32] Shin KC, Oh DK. Production of ginsenoside Rd from ginsenoside Rc by α-L-arabinofuranosidase from *Caldicellulosiruptor saccharolyticus*. J Microbiol Biotechnol. 2013;23(4):483–8. https://doi.org/10.4014/jmb.1211.11012. . 23568202 10.4014/jmb.1211.11012

[33] Lee JH, Hyun YJ, Kim DH. Cloning and characterization of α-L-arabinofuranosidase and bifunctional α-L-arabinopyranosidase/β-D-galactopyranosidase from *Bifidobacterium longum* H-1. J Appl Microbiol. 2011;111(5):1097–107. https://doi.org/10.1111/j.1365-2672.2011.05128.x. . 21851513 10.1111/j.1365-2672.2011.05128.x

[34] Shin HY, Park SY, Sung JH, Kim DH. Purification and characterization of α-L-arabinopyranosidase and α-L-arabinofuranosidase from *Bifidobacterium breve* K-110, a human intestinal anaerobic bacterium metabolizing ginsenoside Rb2 and Rc. Appl Environ Microbiol. 2003;69(12):7116–23. https://doi.org/10.1128/AEM.69.12.7116-7123.2003. . 14660356 10.1128/AEM.69.12.7116-7123.2003 PMC309963

[35] Zhang C, Yu H, Bao Y, An L, Jin F. Purification and characterization of ginsenoside-α-arabinofuranase hydrolyzing ginsenoside Rc into Rd from the fresh root of *Panax ginseng*. Process Biochem. 2002;37(7):793–8. https://doi.org/10.1016/S0032-9592(01)00275-8.

[36] Son JW, Kim HJ, Oh DK. Ginsenoside Rd production from the major ginsenoside Rb1 by β-glucosidase from *Thermus caldophilus*. Biotechnol Lett. 2008;30(4):713–6. https://doi.org/10.1007/s10529-007-9590-4. . 17989924 10.1007/s10529-007-9590-4

[37] Shin KC, Oh DK. Characterization of a novel recombinant β-glucosidase from *Sphingopyxis alaskensis* that specifically hydrolyzes the outer glucose at the C-3 position in protopanaxadiol-type ginsenosides. J Biotechnol. 2014;172:30–7. https://doi.org/10.1016/j.jbiotec.2013.11.026. . 24333127 10.1016/j.jbiotec.2013.11.026

[38] Jin F, Yu H, Fu Y, An DS, Im WT, Lee ST, et al Biotransformation of ginsenosides (ginseng saponins). Int J Biomed Pharm Sci. 2012;6:33–44.

[39] Wang L, Liu QM, Sung BH, An DS, Lee HG, Kim SG, et al Bioconversion of ginsenosides Rb1, Rb2, Rc and Rd by novel β-glucosidase hydrolyzing outer 3-*O* glycoside from *Sphingomonas* sp. 2F2: cloning, expression, and enzyme characterization. J Biotechnol. 2011;156(2):125–33. https://doi.org/10.1016/j.jbiotec.2011.07.024. . 21906640 10.1016/j.jbiotec.2011.07.024

[40] Lee GW, Yoo MH, Shin KC, Kim KR, Kim YS, Lee KW, et al β-Glucosidase from *Penicillium aculeatum* hydrolyzes exo-, 3-*O*-, and 6-*O*-β-glucosides but not 20-*O*-β-glucoside and other glycosides of ginsenosides. Appl Microbiol Biotechnol. 2013;97(14):6315–24. https://doi.org/10.1007/s00253-013-4828-7. . 23504080 10.1007/s00253-013-4828-7

[41] Lee GW, Kim KR, Oh DK. Production of rare ginsenosides (compound Mc, compound Y and aglycon protopanaxadiol) by β-glucosidase from *Dictyoglomus turgidum* that hydrolyzes β-linked, but not α-linked, sugars in ginsenosides. Biotechnol Lett. 2012;34(9):1679–86. https://doi.org/10.1007/s10529-012-0949-9. . 22648684 10.1007/s10529-012-0949-9

[42] Shin KC, Oh HJ, Kim BJ, Oh DK. Complete conversion of major protopanaxadiol ginsenosides to compound K by the combined use of α-L-arabinofuranosidase and β-galactosidase from *Caldicellulosiruptor saccharolyticus* and β-glucosidase from *Sulfolobus acidocaldarius*. J Biotechnol. 2013;167(1):33–40. https://doi.org/10.1016/j.jbiotec.2013.06.003. .22648684. 23774035 10.1016/j.jbiotec.2013.06.003

